# In Situ Coupling Strategy for Anchoring Monodisperse Co_9_S_8_ Nanoparticles on S and N Dual-Doped Graphene as a Bifunctional Electrocatalyst for Rechargeable Zn–Air Battery

**DOI:** 10.1007/s40820-018-0231-3

**Published:** 2019-01-09

**Authors:** Qi Shao, Jiaqi Liu, Qiong Wu, Qiang Li, Heng-guo Wang, Yanhui Li, Qian Duan

**Affiliations:** School of Materials Science and Engineering, Changchun University of Science and Technology, Changchun, 130022 People’s Republic of China

**Keywords:** In situ coupling strategy, Porphyrin derivate, Doped graphene, Metal sulfide, Bifunctional electrocatalyst, Rechargeable Zn–air battery

## Abstract

An in situ coupling strategy to prepare Co_9_S_8_/S and N dual-doped graphene composite (Co_9_S_8_/NSG) has been proposed. The key point of this strategy is the function-oriented design of organic compounds. Herein, cobalt porphyrin derivatives with sulfo groups are employed as not only the coupling agents to form and anchor Co_9_S_8_ on the graphene in situ, but also the heteroatom-doped agent to generate S and N dual-doped graphene. The tight coupling of multiple active sites endows the composite materials with fast electrochemical kinetics and excellent stability for both oxygen reduction reaction (ORR) and oxygen evolution reaction (OER). The obtained electrocatalyst exhibits better activity parameter (Δ*E* = 0.82 V) and smaller Tafel slope (47.7 mV dec^−1^ for ORR and 69.2 mV dec^−1^ for OER) than commercially available Pt/C and RuO_2_. Most importantly, as electrocatalyst for rechargeable Zn–air battery, Co_9_S_8_/NSG displays low charge–discharge voltage gap and outstanding long-term cycle stability over 138 h compared to Pt/C–RuO_2_. To further broaden its application scope, a homemade all-solid-state Zn–air battery is also prepared, which displays good charge–discharge performance and cycle performance. The function-oriented design of N_4_-metallomacrocycle derivatives might open new avenues to strategic construction of high-performance and long-life multifunctional electrocatalysts for wider electrochemical energy applications.

**Electronic supplementary material:**

The online version of this article (10.1007/s40820-018-0231-3) contains supplementary material, which is available to authorized users.

## Highlights

- An effective in situ coupling strategy is proposed to construct Co_9_S_8_ nanoparticles/doped graphene.
- Cobalt porphyrin derivative is employed as both coupling and heteroatom-doped agents.
- The bifunctional oxygen electrocatalyst finds application in rechargeable all-solid-state Zn–air batteries.

**Electronic supplementary material:**

The online version of this article (10.1007/s40820-018-0231-3) contains supplementary material, which is available to authorized users.

## Graphical abstract

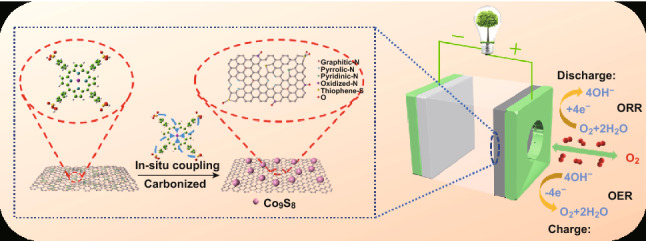

## Introduction

Rechargeable Zn–air battery (ZAB), as one of the most promising power technologies, has attracted significant research interest due to its environment-friendliness, low cost, and high theoretical energy density [1–4]. However, the large voltage gap and poor cycle life have severely hindered its practical application [5]. Therefore, durable bifunctional electrocatalysts for oxygen reduction reaction (ORR) and oxygen evolution reaction (OER) are urgently required to accelerate the recharge rate and overall electrochemical reactions of ZAB [6, 7]. To date, Pt-based materials have been considered state-of-the-art ORR catalysts, while Ir/Ru-based catalysts are considered efficient for OER [8]. However, the prohibitive cost, poor durability, and single function for ORR or OER of these precious-metal-based catalysts are major foundational barriers [4]. An ideal solution to the bottleneck problem is to replace commercial Pt- and Ir/Ru-based catalysts with the highly efficient and durable bifunctional electrocatalysts based on naturally abundant elements [9]. Currently, transition metal sulfides (TMSs) [10, 11], especially Co_9_S_8_ [12, 13], have gained considerable attention due to their nature abundance, environment-friendliness, good durability, and high catalytic activity for both ORR and OER. Unfortunately, their low electronic conductivity has degraded their practical performance. Therefore, it is necessary to employ a highly conductive carbon matrix to anchor the rationally designed TMS nanoparticles.

To this end, graphene has been recognized as an effective matrix due to its high conductivity, chemical stability, and extraordinary specific surface area [14, 15]. Further, doping graphene with heteroatoms (such as N and S) can improve conductivity and provide additional electrocatalytic active sites [16–18]. Therefore, the incorporation of nanostructured TMSs into doped graphene has been intensively studied [12, 13]. However, simple incorporation may result in aggregation of the nanoparticles, thereby hampering exposure of active sites and leading to low catalytic activities. Furthermore, the weak anchors between nanoparticles and graphene cause nanoparticle leaching, resulting in poor durability. Therefore, incorporating N_4_-metallomacrocycles into carbon matrix seems to be a promising approach. On the one hand, the N_4_-metallomacrocycles can act as the coupling agent to anchor nanoparticles [19], thus accomplishing in situ anchoring of small and homogeneously distributed nanoparticles. On the other hand, the tunable structure of N_4_-metallomacrocycles with various heteroatom-containing functional groups endows them with additional functions. These functional groups can be employed as interfacial linkers to link graphene or graphene oxide via aromatic *π*–*π* interactions and reciprocal electrostatic interactions [20], thus realizing heteroatom-doped graphene. Moreover, it is universally accepted that heat-treated N_4_-metallomacrocycles can display high catalytic activity and chemical stability, with Me-N_4_ acting as the catalytic centers for ORR [21]. However, direct synthesis of TMSs through this strategy remains challenging because their synthesis needs additional sulfuration reactions with sulfur or S-containing compounds, which in turn suffer from the shortcomings of using toxic precursors, sophisticated process, and/or the release of poisonous gases. Therefore, it is highly desirable to achieve the function-oriented design of N_4_-metallomacrocycles with S-containing functional groups, which could couple and anchor TMSs nanoparticles on doped graphene in situ as a high-performance bifunctional electrocatalyst for ORR and OER, even ZAB.

In this paper, for the first time, we report a function-oriented design of N_4_-metallomacrocycle derivatives to synthesize Co_9_S_8_/S and N dual-doped graphene composite (Co_9_S_8_/NSG). As a proof-of-concept demonstration, we used cobalt(II) 5,10,15,20-tetra-(4-sulfonatophenyl) porphyrin (TSPPCo) as not only the coupling agent to form and anchor Co_9_S_8_ on the graphene in situ, but also the heteroatom-doped agent to form S and N dual-doped graphene in situ. Benefiting from the function-oriented design and unique structure, the Co_9_S_8_/NSG exhibits high catalytic activity and outstanding stability for ORR and OER. To investigate its practical applications, a homemade all-solid-state ZAB is built based on our bifunctional electrocatalysts, which displays high performance and excellent long cycle life.

## Experimental Section

### Synthesis of Catalyst

Graphene oxide solution (4 g, 2.5 wt%), TSPPCo (0.05, 0.1, and 0.15 g), and 10 mL water were added to a 50-mL Teflon-lined autoclave and stored at 180 °C for 24 h. After cooling to room temperature, it was freeze-dried under vacuum, followed by calcination at 600, 700, and 800 °C for 2 h in N_2_, respectively. The obtained products were labeled as Co_9_S_8_/NSG-600, Co_9_S_8_/NSG-700, and Co_9_S_8_/NSG-800, respectively. Moreover, GO with different loading contents of TSPPCo (0.05, 0.1, and 0.15 g) were denoted as Co_9_S_8_/NSG-700-0.5, Co_9_S_8_/NSG-700, and Co_9_S_8_/NSG-700-1.5, respectively. Co_9_S_8_/C-700 was synthesized by a method similar to that used for Co_9_S_8_/NSG without the presence of GO, and NSG-700 was obtained by leaching the pyrolyzed product in HCl aqueous solution (0.1 M) for 8 h to remove Co_9_S_8_.

### Electrochemical Measurements

All the electrochemical measurements of the electrocatalysts for ORR/OER were taken on a CS350 electrochemical workstation in the corresponding electrolytic solution using a standard three-electrode cell, in which a rotating disk electrode of diameter 5.0 mm (RDE, Pine Research Instrument, USA) served as the working electrode, Pt-foil as the counter electrode, and saturated calomel electrode (SCE) as the reference electrode.

To evaluate the ORR and OER performances, cyclic voltammetry (CV) was performed in N_2_- or O_2_-saturated solution with a scan rate of 50 mV s^−1^. Linear sweep voltammetry (LSV) measurements for ORR were taken at different speeds from 400 to 1600 rpm in an O_2_-saturated solution with a sweep rate of 10 mV s^−1^ without using iR-correction. LSV measurements for OER were also taken using the same three-electrode cell in O_2_-saturated 1 M KOH solution with a scan rate of 5 mV s^−1^ with iR-correction. Before all the electrochemical characterizations, the continuous sweep of the corresponding voltage range was measured until a steady voltammogram curve was obtained.

The durability tests of the ORR/OER electrocatalysts were both performed using chronoamperometric (*i* − *t*) measurement in O_2_-saturated corresponding solutions at a rotation rate of 1600 rpm, while 10 vol% methanol was added for demonstrating methanol tolerance during ORR.

### Zn–Air Battery Assembly and Measurements

The air–electrode used for ZAB was composed of carbon paper as the catalyst-loaded layer (1 mg cm^−2^) facing the water side and the gas diffusion layer facing the air side. A zinc plate was used as the anode, while 6 M KOH containing 0.2 M Zn(Ac)_2_ was used as the electrolyte for ZAB. The effective area of the catalyst-loaded layer and zinc plate is controlled to 1 cm^2^.

The homemade all-solid-state ZAB was also fabricated using zinc foil as anode and the catalyst-loaded carbon paper as the air-electrode; however, a solid polymer electrolyte is used as a separator for the battery. The solid polymer electrolyte was prepared by the following steps. First, polyvinyl alcohol powder (4.5 g) was dissolved in 0.1 M KOH (40 mL) containing 0.02 M Zn(Ac)_2_ and then stirred at 90 °C for 2 h. The solution was then poured into a culture dish and dried at 55 °C to form a solid polymer film.

All the electrochemical tests of ZAB were conducted on the CS350 electrochemical workstation in ambient air. The galvanodynamic charge–discharge profiles were obtained via LSV (5 mV s^−1^). The cycling curves were obtained using 400 s for each cycle.

## Results and Discussion

**Fig. 1:**
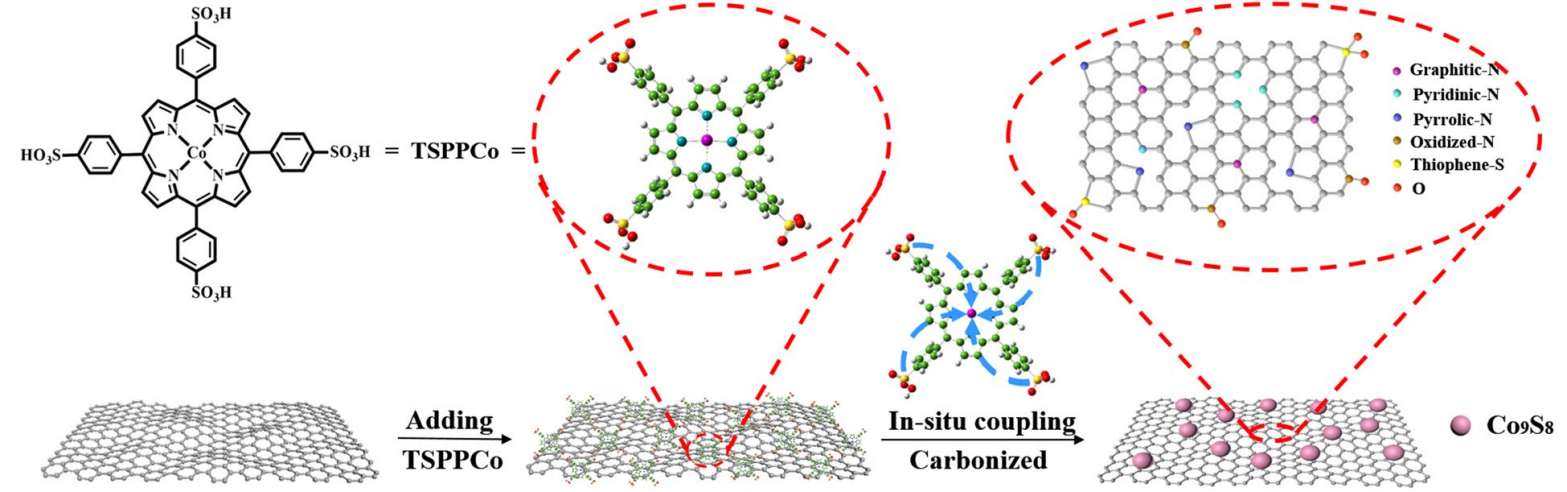
Schematic illustration of the synthesis of Co_9_S_8_/NSG

Figure 1 schematically illustrates the fabrication process of Co_9_S_8_/NSG. First, TSPP molecules were synthesized by sulfonating TPP. (The purity and identity of TPP and TSPP were verified by ^1^H NMR spectroscopy, as shown in Figs. S1 and S2.) After that, the TSPPCo molecules were synthesized by coordinating TSPP molecules with Co^2+^ ions, which were subsequently mixed with the GO solution. Herein, on the one hand, the sulfonic groups could endow water solubility of TSPPCo to make the mixture with GO solution more uniform, enabling the anchoring of TSPPCo molecules on the surface of the GO sheets via *π*–*π* interactions. On the other hand, the axially covalent connection of TSPPCo with graphene would prevent TSPPCo molecules from deformation and aggregation during the subsequent calcination [22]. Finally, carbonization was applied to obtain Co_9_S_8_/NSG. It is necessary to point out that TSPPCo acts as the single source of active sites (N, S, Co–N–C, and Co_9_S_8_) and plays the dual role of heteroatom-doped source and coupling agent. It could not only obtain the multi-heteroatom-doped graphene, but also generate the Co_9_S_8_ by in situ coupling.

**Fig. 2:**
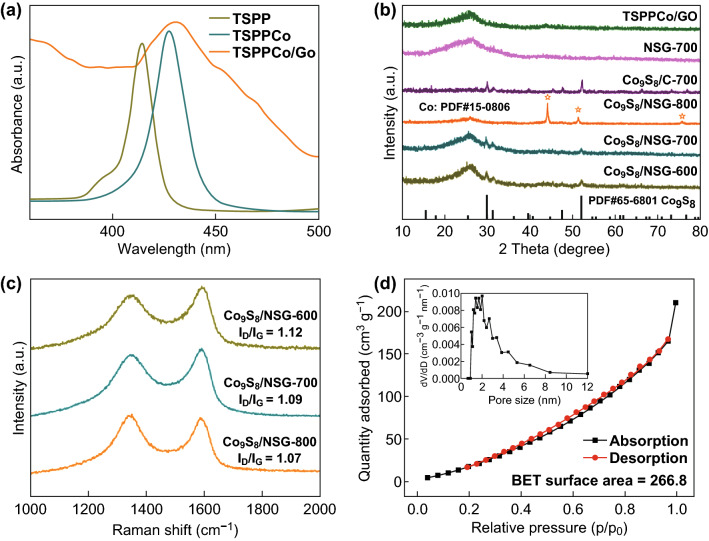
**a** UV–Vis absorption spectra of TSPP, TSPPCo, and TSPPCo/GO. **b** XRD patterns and **c** Raman spectra of the different samples. **d** N_2_ adsorption–desorption isotherms and the pore size distribution (inset) of Co_9_S_8_/NSG-700

UV–Vis absorption spectroscopy was used to confirm the synthesis of TSPPCo/GO (Figs. 2a and S3). The TSPP solution exhibited five peaks, corresponding to the intense Soret band at 412 nm and four weak Q-bands at 515, 551, 581, and 633 nm [23]. After coordination with Co^2+^, the TSPPCo solution exhibited a characteristic absorption peak centered at 426 nm from the intense Soret band and a weak peak at 539 nm from the Q-bands. Thus, not only a redshift of the Soret band could be discerned, but also the number of Q-bands was reduced, which may be ascribed to the increasing symmetry of the molecules when the metal ion coordinates with the N atoms [24]. Both the Q-band and the Soret bands showed redshift after anchoring the TSPPCo on GO, indicating successful formation of TSPPCo and GO composite [25]. As shown in the FTIR spectra, the distinct characteristic peaks of N–H at ~ 3316 and ~ 967 cm^−1^ [26], those of aromatic rings at ~ 1399 and ~ 1193 cm^−1^, and those of −SO_3_ at ~ 1039 and ~ 637 cm^−1^ indicate the successful synthesis of TSPP (Fig. S4a). In addition, no peaks corresponding to N–H could be observed for TSPPCo, which confirms the successful incorporation of metal into TSPP. For TSPPCo/GO, both the distinct characteristic peaks of TSPPCo and the GO peaks of C–O at ~ 1174 cm^−1^ and C=C at ~ 1582 cm^−1^ were observed (Fig. S4b). After carbonization, no peaks corresponding to TSPPCo were observed for Co_9_S_8_/NSG-700, confirming the decomposition of TSPPCo during carbonization. The XRD patterns of TSPPCo/GO (Fig. 2b) showed broad diffraction peaks at ~ 24.0° and 43.3°, which could be related to the (002) and (101) diffractions of disordered carbon [27]. After carbonization, Co_9_S_8_/NSG-700 and Co_9_S_8_/NSG-600 exhibited not only the peaks of disordered carbon, but also the intense diffraction peaks of Co_9_S_8_ (JCPDS card no. 65-6801). According to Scherrer formula, the average diameter of Co_9_S_8_ was around 15 nm. Co_9_S_8_/NSG-800 displayed well-defined diffraction peaks of crystalline Co, demonstrating the difficulty of forming Co_9_S_8_ at calcination temperatures above 700 °C using this strategy. For the Co_9_S_8_/C-700 sample, only the peaks of Co_9_S_8_ could be observed, suggesting that single TSPPCo could also form Co_9_S_8_. Besides, no diffraction peak of Co_9_S_8_ could be detected in NSG-700, suggesting that Co_9_S_8_ was completely removed. As shown in the Raman spectra (Fig. 2c), the ratio of *I*_D_/*I*_G_ of Co_9_S_8_/NSG-600 (1.12) was higher than those of Co_9_S_8_/NSG-700 (1.09) and Co_9_S_8_/NSG-800 (1.07), indicating that the degree of disordered structure decreased with increasing carbonization temperature [28, 29]. In addition, the intensity of the D band is lower than that of the G band, manifesting that Co_9_S_8_/NSG was partially graphitized. Moreover, the N_2_ adsorption–desorption isotherms and the pore size distribution of Co_9_S_8_/NSG-700 (Fig. 2d) show that Co_9_S_8_/NSG-700 has a significant specific surface area (SSA) of 266.8 m^2^ g^−1^ and pore sizes ranging from 1 to 8 nm. On the other hand, the SSAs of Co_9_S_8_/NSG-600 (248.0 m^2^ g^−1^) and Co_9_S_8_/NSG-800 (281.2 m^2^ g^−1^) were similar to that of Co_9_S_8_/NSG-700, while that of NSG-700 (237.4 m^2^ g^−1^) was lower than that of Co_9_S_8_/NSG-700 (Fig. S5). Thermogravimetry was carried out to evaluate the percentage of Co_9_S_8_ in the composite, from which the weight percentage of Co_9_S_8_ was calculated to be 36% (Fig. S6).

**Fig. 3:**
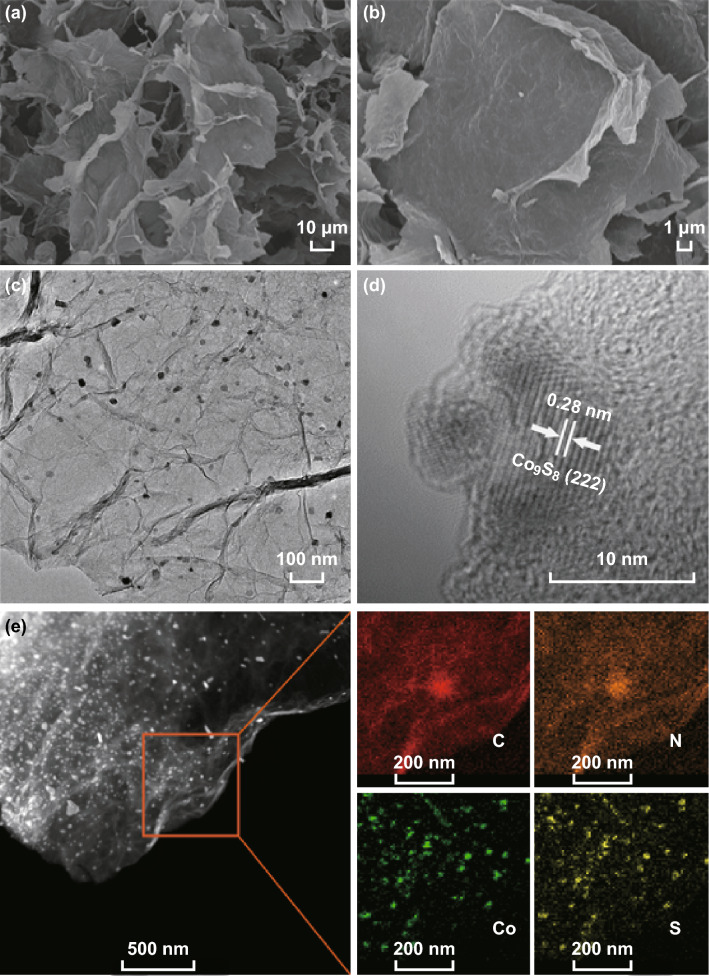
**a** Low- and **b** high-resolution SEM images of Co_9_S_8_/NSG-700. **c** TEM image of Co_9_S_8_/NSG-700. **d** HRTEM image of Co_9_S_8_/NSG-700. **e** TEM image and elemental mapping of carbon, nitrogen, cobalt, and sulfur

Subsequently, scanning and transmission electron microscopy (SEM and TEM, respectively) images were obtained to observe the morphology of Co_9_S_8_/NSG, which showed two-dimensional (2D) thin graphene sheets (Figs. 3a–b and S7). In contrast, Co_9_S_8_/C-700 showed aggregation instead of the 2D GO nanosheets (Fig. S8), which confirms the important role of the GO matrix. Further information about the Co_9_S_8_/NSG-700, obtained from TEM images (Fig. 3c), demonstrates that Co_9_S_8_ nanocrystals were homogeneously monodispersed on GO sheets without aggregation. The average diameter of Co_9_S_8_ was calculated to be ~ 15 nm from the particle size distribution obtained from the TEM image (Fig. S9), in accordance with the result obtained from XRD analysis. The HRTEM image showed that the lattice fringe with a distance of 0.28 nm was related to the (222) crystal face of Co_9_S_8_ (Fig. 3d). More detailed information was obtained from the TEM image and from elemental mapping. Figure 3e shows the homogeneous dispersion of C, N, Co, and S, demonstrating the homogeneous dispersion of Co_9_S_8_ on the surface of the S and N dual-doped graphene matrix.

**Fig. 4:**
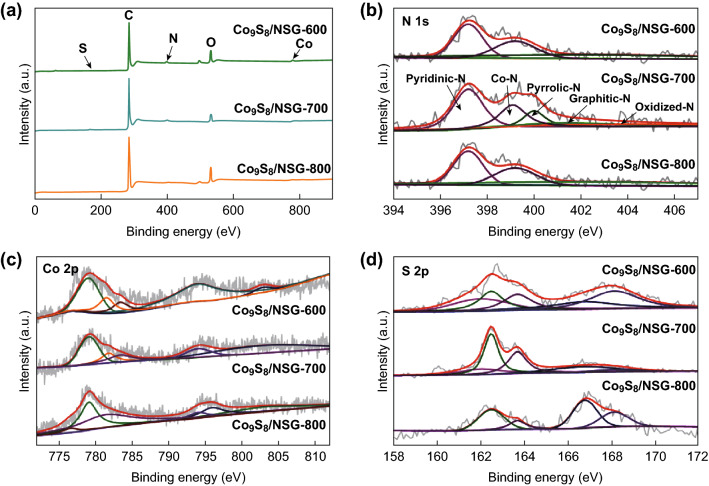
**a** XPS survey, and high-resolution XPS spectra of **b** N 1 s, **c** Co 2p, and **d** S 2p of Co_9_S_8_/NSG-600, Co_9_S_8_/NSG-700, and Co_9_S_8_/NSG-800

X-ray photoelectron spectroscopy (XPS) was performed to obtain more information about Co_9_S_8_/NSG. Figure 4a shows the presence of S, C, N, O, and Co in various samples. The spectra of N 1 s (Fig. 4b) are resolved into five peaks that can be related to pyridinic-N (397.2 eV), Co–N (399.4 eV), pyrrolic-N (400 eV), graphitic-N (401 eV), and oxidized-N (402.7 eV) [30–32]. Pyridinic-N accounted for most of the doped nitrogen atoms, which could improve the onset potential for ORR [33]. The high-resolution Co 2p XPS spectra (Fig. 4c) show that the peak at 783.4 eV is related to Co–S, the peak at 779.1 eV is assigned to Co–N, the peak at 781.5 eV is related to Co 2*p*_3/2_, and the peaks at 794 and 802.9 eV correspond to Co 2*p*_1/2_ [34–37]. The appearance of Co 2*p*_1/2_ and Co 2*p*_3/2_ may be due to the surface oxidation of metallic Co in air, which would promote the rate of OER [38]. Besides these, Co_9_S_8_/NSG-800 exhibited peaks at 795.9, 781.4, and 776.6 eV, corresponding to Co (0). In the S 2p XPS spectra (Fig. 4d), there are five peaks centered at 162, 162.5, 163.7, 166.8, and 168.15 eV, corresponding to Co–S, S 2*p*_1/2_, S 2*p*_3/2_, C=S, and S–O, respectively [27, 39, 40]. It is well known that the sulfur species could induce the redistribution of “electron spin” [41]; therefore, the presence of sulfur in the Co_9_S_8_/NSG-700 would contribute to the electrocatalytic activity. It is also worth mentioning that Co_9_S_8_/NSG-700 contained the highest total content of Co–N and pyridinic-N, which may endow it with good ORR activity.

**Fig. 5:**
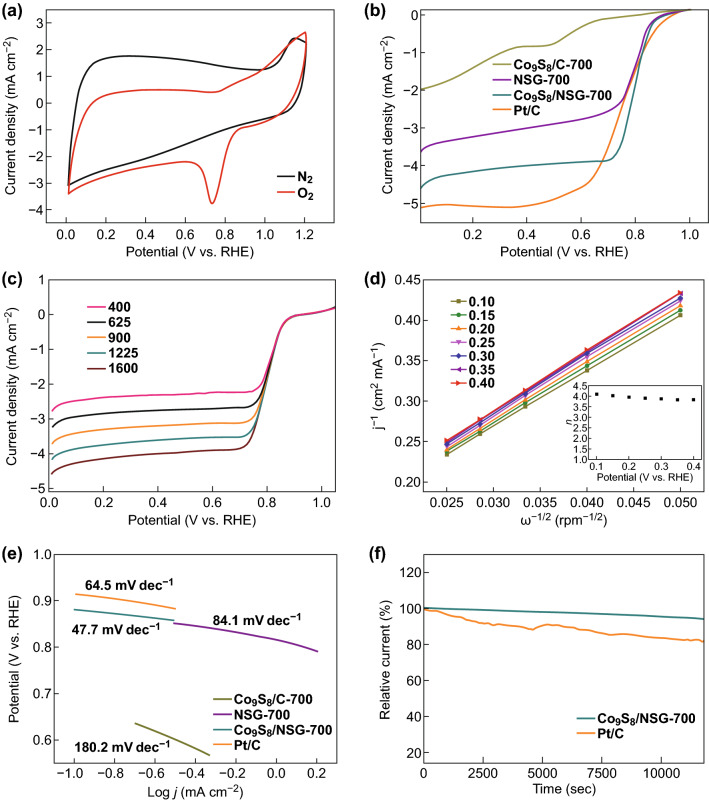
ORR performance of Co_9_S_8_/NSG-700 in 0.1 M KOH. **a** CV curves of Co_9_S_8_/NSG-700 in N_2_-saturated and O_2_-saturated solutions. **b** LSV curves of Co_9_S_8_/C-700, NSG-700, Co_9_S_8_/NSG-700, and Pt/C at 1600 rpm. **c** LSV curves of Co_9_S_8_/NSG-700 at different rotating rates. **d** K–L plots and the electron transfer number (inset) obtained from RDE results of Co_9_S_8_/NSG-700. **e** Tafel plots of the samples. **f** Current–time (*i* − *t*) chronoamperometric response of Co_9_S_8_/NSG-700 and Pt/C in O_2_-saturated 0.1 M KOH

Based on the unique structure and composition mentioned above, the ORR activity of the as-obtained Co_9_S_8_/NSGs was investigated. Comparison of the LSV curves of the different samples (Fig. S10) revealed that carbonization temperature and Co_9_S_8_ content are both critical parameters for ORR activity. Co_9_S_8_/NSG-700 showed the best ORR activity in terms of onset potential (*E*_O_) and/or current density (*J*_L_) by optimizing the carbonization temperature (600, 700, or 800 °C) and the loading content of TSPPCo (0.05, 0.1 or 0.15 g). The CV curves of Co_9_S_8_/NSG exhibited no cathodic peak in N_2_-saturated solution, while a pronounced cathodic peak at 0.74 V was observed in O_2_-saturated solution (Fig. 5a). As shown in Fig. 5b, the Co_9_S_8_/NSG-700 exhibited *E*_O_ of 0.92 V, comparable to that of commercial Pt/C (0.94 V), good half-wave potential (*E*_1/2_) of 0.79 V, and limited *J*_L_ of 4.59 mA cm^−2^. In contrast, the NSG exhibited lower *E*_O_ (0.90 V) and *J*_L_ (3.6 mA cm^−2^), which could prove that in situ coupling and anchoring of Co_9_S_8_ in S and N dual-doped graphene could enhance ORR activity. Moreover, Co_9_S_8_/C-700 showed significantly poor *E*_O_ (0.88 V) and limited *J*_L_ (1.97 mA cm^−2^) compared with that of Co_9_S_8_/NSG-700, probably due to the absence of the graphene matrix. On the one hand, the S and N dual-doped graphene could not only improve conductivity, but also provide additional active sites. On the other hand, the Co_9_S_8_ molecules could be anchored on the graphene in situ, which suppressed the aggregation of Co_9_S_8_ nanocrystals, thus improving ORR activity. The ORR polarization curves of Co_9_S_8_/NSG were recorded at different rotating speeds (Fig. 5c), indicating that *J*_L_ increased gradually with increasing rotating speed due to the shorter diffusion distance of oxygen at higher speeds. Moreover, the Koutecky–Levich (K–L) plots of Co_9_S_8_/NSG-700 exhibited excellent linearity and parallelism (Fig. 5d), revealing first-order reaction kinetics [42]. The electron transfer number was calculated to be 3.8–4.0, revealing a four-electron transfer pathway [43]. Co_9_S_8_/NSG-600 and Co_9_S_8_/NSG-800 also showed similar LSV curves, corresponding K–L plots, and electron transfer numbers (Fig. S11). Moreover, the significant ORR performance of Co_9_S_8_/NSG-700 was also confirmed by the smaller Tafel slope (47.7 mV dec^−1^), compared with that of Pt/C (64.5 mV dec^−1^) and other obtained materials (Fig. 5e). Besides ORR activity, the stability of the Co_9_S_8_/NSG-700 was essential for practical applications. The durability test was performed using *i* − *t* chronoamperometric response. As shown in Fig. 5f, 95% of the initial current was retained for Co_9_S_8_/NSG-700 after 12,000 s, while only 82% was retained for Pt/C.

**Fig. 6:**
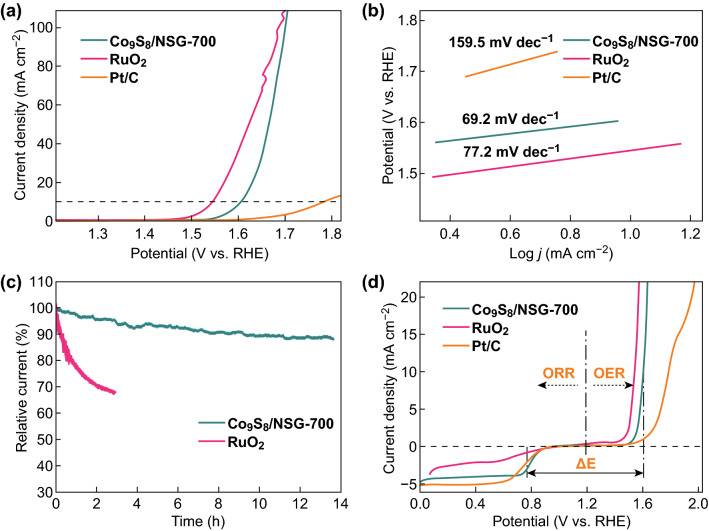
OER performance of Co_9_S_8_/NSG-700 in 1 M KOH. **a** LSV curves and **b** Tafel plots of Co_9_S_8_/NSG-700, RuO_2_, and Pt/C. **c** Current–time (*i* − *t*) chronoamperometric response of Co_9_S_8_/NSG-700 and RuO_2_. **d** Combined ORR/OER LSV curves of Co_9_S_8_/NSG-700, RuO_2_, and Pt/C

Along with ORR, OER is important for various renewable power technologies [44], especially ZAB [3]. To this end, the OER catalytic activity of Co_9_S_8_/NSG-700 was explored. As shown in Fig. 6a, Co_9_S_8_/NSG-700 displays a potential of 1.61 V to achieve 10 mA cm^−2^, which is 50 mV higher than that for RuO_2_ but 160 mV lower than that for Pt/C. Co_9_S_8_/NSG-700 exhibits a much smaller Tafel slope of 69.2 mV dec^−1^ than those of RuO_2_ (77.2 mV dec^−1^) and Pt/C (159.5 mV dec^−1^), indicating the faster kinetic process (Fig. 6b). In addition, the durability tests were performed using *i* − *t* chronoamperometric technique for 13.5 h (Fig. 6c). Approximately 88% of the initial current was retained for Co_9_S_8_/NSG-700, while only ~ 67% was retained for RuO_2_ after 12,000 s. Moreover, the LSV curves only display a decay of 8 mV after a 2000-cycle CV scan (Fig. S12), revealing the superior stability of Co_9_S_8_/NSG-700 for OER. The structure and chemical constitution of Co_9_S_8_/NSG-700 were also investigated after the OER test. SEM images show that 2D graphene sheets were retained after OER (Fig. S13a), and TEM images show that the nanocrystals remained homogeneously monodispersed on the surface of the GO sheets without any obvious change (Fig. S13b). XPS analysis was also performed after OER test (Fig. S14). The types of N in the high-resolution N 1*s* XPS spectra were found to be the same as those of the catalyst before the test (Fig. S14a). Interestingly, the detailed scan of Co 2p showed the presence of CoOOH (781.7 and 789.9 eV) and cobalt oxides (785.5, 796.17, 798.73, and 803.02 eV) (Fig. S14b) [39, 45]. In the S 2p XPS spectra (Fig. S14c), there were two peaks centered at 164.0 and 165.3 eV corresponding to S=C, while two peaks at 168.4 and 169.6 eV corresponded to S–O [45]. Further, comparison of the contents of elemental C, O, N, S, and Co in the Co_9_S_8_/NSG-700 before and after OER test (Table S1) revealed that C, N, S, and Co contents in Co_9_S_8_/NSG-700 exhibit almost no fluctuation and the O content increases, probably due to the formation of cobalt oxides and CoOOH. Furthermore, the LSV curves of Co_9_S_8_/NSG-700, RuO_2_, and Pt/C were combined to evaluate the ORR/OER bifunctional properties (Fig. 6d). The bifunctional properties could be judged by the variance in OER/ORR potential (Δ*E *= *E*_j = 10_ − *E*_1/2_; *E*_j = 10_ is the OER potential required to achieve 10 mA cm^−2^, while *E*_1/2_ is the half-wave potential of ORR). Obviously, the lower Δ*E* value indicated better bifunctional activity. It should be emphasized that Co_9_S_8_/NSG-700 displays much lower Δ*E* (0.82 V) than RuO_2_ (0.91 V) and Pt/C (1.05 V). Overall, our bifunctional electrocatalysts showed catalytic performances comparable to reported results (Table S2).

It is worth mentioning that the outstanding electrochemical performance and stability of Co_9_S_8_/NSG-700 could be attributed to the unique characteristics, which could be elaborated as follows. On the one hand, the graphene matrix composed of nanosheets can provide large surface area, thus increasing the exposure and adsorption at more active sites on the catalyst surface. Moreover, the S and N dual-doped graphene can endow the catalyst with high conductivity and additional electrocatalytic active sites. On the other hand, the abundant active sites, including N, S, Co–N, and Co_9_S_8_, derived from the TSPPCo precursor, could promote the ORR/OER activity, and the strong binding interaction derived from the in situ coupling and anchoring of Co_9_S_8_ on the graphene could prevent the leaching and aggregation of the Co_9_S_8_ nanoparticles. As a result, benefitting from the advantageous properties of large surface area, high conductivity, and tight coupling, Co_9_S_8_/NSG displayed high ORR/OER activity and good stability.

**Fig. 7:**
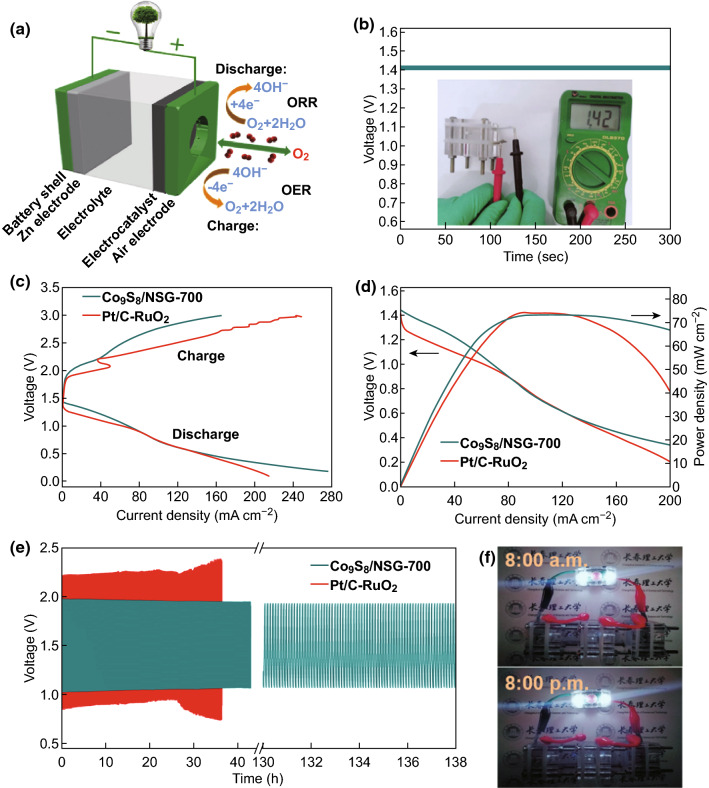
**a** Schematic illustration of the assembled rechargeable Zn–air battery. **b** Open-circuit plots of Co_9_S_8_/NSG-700 (inset: photograph of the open-circuit potential). **c** Galvanodynamic charge–discharge profiles of Co_9_S_8_/NSG-700 and Pt/C–RuO_2_. **d** Galvanodynamic discharge curve profiles and corresponding power density curves of Co_9_S_8_/NSG-700 and Pt/C–RuO_2_. **e** Cycling curves of the Co_9_S_8_/NSG-700 and Pt/C–RuO_2_ at a current density of 10 mA cm^−2^. **f** Photographs of an LED bike lamp powered by three Zn–air batteries of Co_9_S_8_/NSG-700 catalysts before and after 12 h (8:00 a.m. and 8:00 p.m., respectively)

Inspired by the outstanding ORR/OER performance and stability, ZAB was built using Co_9_S_8_/NSG-700 (1 mg cm^−2^) as the air–cathode catalyst and zinc plate as the anode (Fig. 7a). ZAB using Pt/C–RuO_2_ (1:1) as the air–cathode catalyst was also constructed for comparison. The assembled battery using Co_9_S_8_/NSG-700 showed an open-circuit voltage of ~ 1.42 V (Fig. 7b), higher than that for Pt/C–RuO_2_ (1.40 V) (Fig. S15). The galvanodynamic charge–discharge profiles for ZAB (Fig. 7c) revealed that ZAB using Co_9_S_8_/NSG-700 had higher maximal current density (274 mA cm^−2^) than that using Pt/C–RuO_2_ (214 mA cm^−2^). Moreover, the maximal peak power density of the battery using Co_9_S_8_/NSG-700 was calculated to be 72.14 mW cm^−2^, comparable to that of the battery using Pt/C–RuO_2_ (74.3 mW cm^−2^) (Fig. 7d). Furthermore, the assembled battery displayed a low charge–discharge voltage gap of 0.86 V with no voltage change observed in the galvanostatic charge–discharge cycling curves of Co_9_S_8_/NSG-700 after cycling for 138 h at 10 mA cm^−2^ (Fig. 7e). In comparison, Pt/C–RuO_2_ displayed lower charge voltage and higher discharge voltage with a significant deterioration after cycling for 26 h, indicating the outstanding durability of Co_9_S_8_/NSG-700. Interestingly, only three such assembled batteries can operate a light-emitting diode (LED) bike lamp over 12 h (Fig. 7f), which is a more direct and easier verification of the excellent robustness of the battery. Interestingly, the performance of the ZAB using Co_9_S_8_/NSG-700 as catalyst was comparable with reported results (Table S3).

**Fig. 8:**
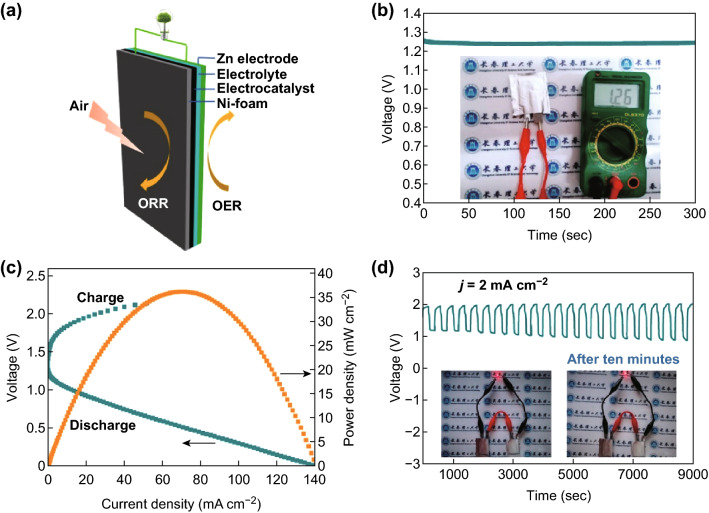
**a** Schematic illustration of the assembled homemade all-solid-state batteries. **b** Open-circuit plots (inset: photograph of open-circuit potential). **c** Galvanodynamic charge–discharge profiles and corresponding power density curves. **d** Cycling curves of the batteries at a current density of 2 mA cm^−2^ (inset: photographs of an LED powered by two all-solid-state Zn–air batteries before and after 10 min)

To further broaden the practical applicability and prospects of Co_9_S_8_/NSG-700, a homemade all-solid-state ZAB with a small size of 2 × 3 cm^2^ was integrated (Fig. 8a). Surprisingly, the assembled battery displayed an open-circuit voltage as high as 1.26 V (Fig. 8b), a maximal current density of 140 mA cm^−2^, and a peak power density of 36.2 mW cm^−2^ (Fig. 8c). Furthermore, when cycled at 2 mA cm^−2^, the all-solid-state battery produced a low initial charge–discharge voltage gap of 0.75 V (charge potential of 1.93 V and discharge potential of 1.20 V), without any prominent changes after 9000 s. Interestingly, only two miniature batteries were needed to light a high-voltage LED, which operates at a minimum voltage of 2.0 V. (Fig. 8d).

## Conclusions

In summary, the novel and effective strategy of using N_4_-metallomacrocycles, with S-containing functional groups, as both the single-source precursor and the coupling agent, is applied to the in situ formation and anchoring of Co_9_S_8_ nanocrystals on the doped graphene. It is worth mentioning that Co_9_S_8_ can be synthesized via this strategy without using additional sulfur or S-containing compounds, thus avoiding the requirement of toxic precursors, sophisticated process, and/or the release of poisonous gases. More importantly, owing to the enhanced conductivity of the S and N dual-doped graphene, the ultrafine Co_9_S_8_ nanocrystals, and in situ coupling interaction, the as-obtained Co_9_S_8_/NSG-700 displayed significant catalytic activity and stability for ORR/OER. Furthermore, as the air-electrode catalyst for ZAB, even all-solid-state ZAB, Co_9_S_8_/NSG-700 exhibited good performance and good stability. Therefore, we believe that the function-oriented design of N_4_-metallomacrocycles, with S-containing functional groups, is versatile and effective for the synthesis of other electrocatalysts for wider practical applications.

## Electronic supplementary material

Below is the link to the electronic supplementary material.

Supplementary material 1 (PDF 1176 kb)
