# Supplementary material for: In Situ Coupling Strategy for Anchoring Monodisperse Co9S8 Nanoparticles on S and N Dual-Doped Graphene as a Bifunctional Electrocatalyst for Rechargeable Zn–Air Battery
Source: Nanomicro Lett. 2019 Jan 9;11:4. doi: 10.1007/s40820-018-0231-3 (PMC7770932; doi:10.1007/s40820-018-0231-3)
Supplement: Supplementary file 1 — Supplementary material 1 (PDF 1176 kb) [file 40820_2018_231_MOESM1_ESM.pdf]

Supporting Information for

In-Situ Coupling Strategy for Anchoring Monodisperse Co_9S_8 Nanoparticles on S and N Dual-Doped Graphene as a Bifunctional Electrocatalyst for Rechargeable Zn-Air Battery

Qi Shao[1], Jiaqi Liu[1], Qiong Wu[1], Qiang Li[1], Heng-guo Wang[1,*], Yanhui Li[1], Qian Duan[1,*]

School of Materials Science and Engineering, Changchun University of Science and Technology, Changchun 130022, People's Republic of China

*****Corresponding authors. E-mail: wanghengguo@cust.edu.cn (H.G. Wang), duanqian88@hotmail.com (Q. Duan)

Tel: +86-431-85583176

S1 Experimental Method

S1.1 Materials

All chemical reagents (including $CoCl_2\cdot 4H_2O$, ethanol, KOH, methanol, polyvinyl alcohol) were purchased from reliable sources (Aladdin Industrial Co., Shanghai; Sinopharm Chem. reagent Co. Ltd, China; Sigma Andrich) and used as received. All the chemicals were analytical grade in purity. Graphene oxide (GO) was synthesized by a modified Hummers Method [S1]. De-ionized water was obtained from an ultra-pure purification system. The zinc foil (99.98% metal basis) was obtained from Alfa Aesar and the light emitting diode (LED) (5 mm size, ~3 V), LED bike lamp (45×20 mm, 4.2 V) and LED scroll displaying screen (93 mm×32 mm, 4.7 V) were obtained from the local supplier.

S1.2 Synthesis of 5, 10, 15, 20-Tetrakis (4-sodiosulfophenyl)-21H, 23H-Porphyrin (TSPP)

5, 10, 15, 20-Tetraphenyl-21H, 23H-porphyrin (TPP) was synthesized following the Adlers Method [S2]. TPP (0.5 g, 0.8 mmol) was dissolved in H_2SO_4 (17 mL, 0.32 mol) under reflux and heating conditions (120 °C) for 2h. After cooling to room temperature, the reaction mixture was poured into 300 mL deionized water and regulated the pH to 7-8 by using NaOH. Thereafter, the as-obtained solution was concentrated and filtered to remove the Na_2SO_4. Subsequently, the resulted solution was added into methanol and filtrated for several times to remove the precipitated Na_2SO_4. Finally, the crude compound was further purified by recrystallization from methanol and acetone for three times to obtain the purified TSPP.

S1.3 Synthesis of Cobalt (II) 5, 10, 15, 20-tetrakis (4-sodiosulfophenyl)-21H, 23H-porphyrin (TSPPCo)

TSPP (0.2 g, 0.2 mmol) and $CoCl_2·4H_2O$ (0.2 g, 1 mmol) were dissolved in 150 mL deionized water and heated to reflux for 4 h. After that, the mixture solution was concentrated and dissolved in methanol. At last, the resulted product was dried under vacuum for 12 h to obtain the TSPPCo.

S1.4 Characterization

The microstructures of the nanomaterials were observed by scanning electron microscopy (SEM Hitachi S-4800) and transmission electron microscope (TEM) recorded on a Tecnai G2 operating at 200 kV. The crystal phases were evaluated by X-ray diffraction (XRD) patterns recorded on a Rigaku-Dmax 2500 diffractometer with Cu Ka radiation. FTIR measurements performed on a Bruker IFS 66V/S spectrometer using KBr pellets. X-ray photoelectron spectroscopy (XPS) analysis conducted with ESCALAB MK II X-ray instrument was used to analyze the composition of the nanomaterials. Raman spectra were collected with a Renishaw 2000 model confocal microscopy Raman spectrometer.

S1.5 Electrochemical Measurements

The recorded potentials versus SCE were converted to a RHE scale based on the Nernst equation ($E_{RHE}=E_{SCE}+0.241+0.059pH$). To prepare the working electrode, 5 mg of Co_9S_8/NSG-700 was ultrasonically dispersed in ethanol (1 mL) with Nafion solution (50 μL) to generate a uniform ink. 10 μL of the catalyst slurry was dropped onto the surface of the electrode and then dried at the room temperature for the measurements of ORR/OER.

According to the LSV curves of ORR at the different potentials, the electron transfer number (n) was calculated according to the Koutecky-Levich (K-L) equations:

$$\frac{1}{J}=\frac{1}{J_D}+\frac{1}{J_K}=\frac{1}{B\omega^{1/2}}+\frac{1}{nFkC_{O_2}}$$

$$\mathrm{B}=0.62\mathrm{nFC_{O_2}}\left(\mathrm{D_{O_2}}\right)^{2/3}\mathrm{v}^{-1/6}$$

Where J is the measured current density using RDE, while J_D and J_K are the diffusion- and kinetic-limiting current density, respectively. n is the number of transferred-electron per oxygen molecule and F is the Faraday constant (96,485 C mol^{-1}). In addition, ω reflects the rotation rate and k is the electron transfer rate constant. Meanwhile, B represents the slope of the following equation. Moreover, C_{O_2} is the bulk concentration (1.1×10^{-3} mol cm^{-3} for 0.5 M H_2SO_4 aqueous solution and 1.2×10^{-3} mol cm^{-3} for 0.1 M KOH aqueous solution), while the D_{O_2} is the diffusion coefficient (1.4×10^{-5} cm^2 s^{-1} for 0.5 M H_2SO_4 solution and 1.9×10^{-5} cm^2 s^{-1} for 0.1 M KOH solution). Besides, v is the kinetic viscosity of solution (0.01 cm^2 s^{-1} for both 0.5 M H_2SO_4 solution and 0.1 M KOH solution).

S2 Supplemental Figures

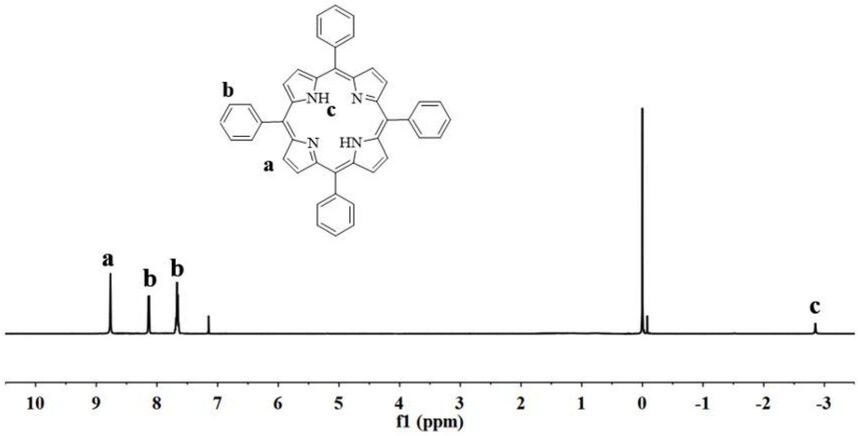

Fig. S1 The ^{1}H-NMR image of TPP

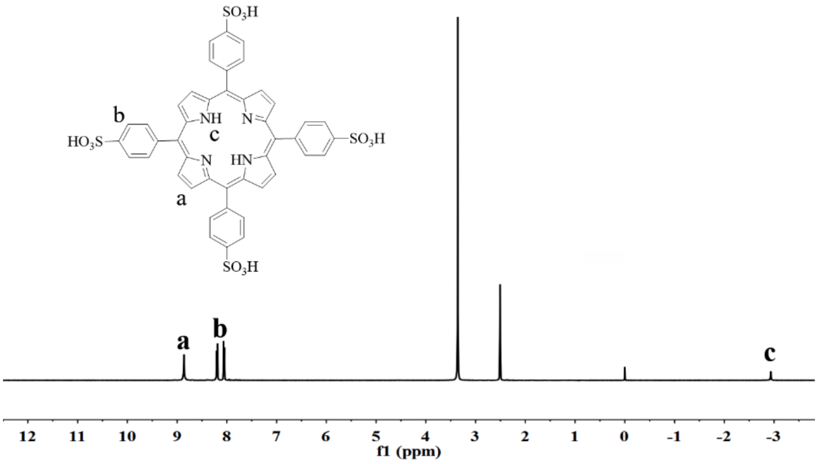

Fig. S2 The ^{1}H-NMR image of TSPP

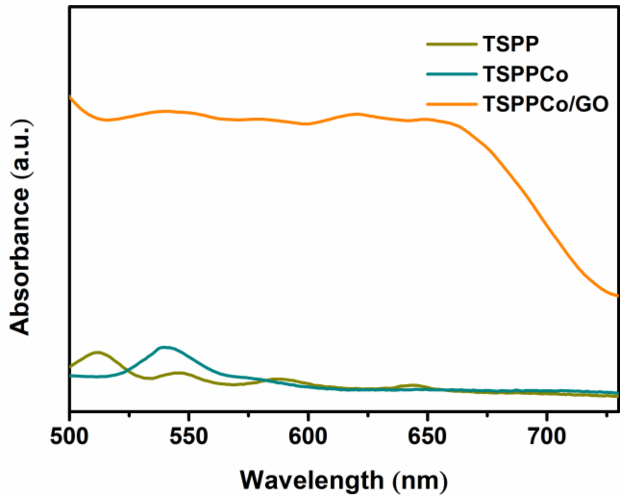

Fig. S3 UV/Vis absorption spectra of TSPP, TSPPCo and TSPPCo/GO

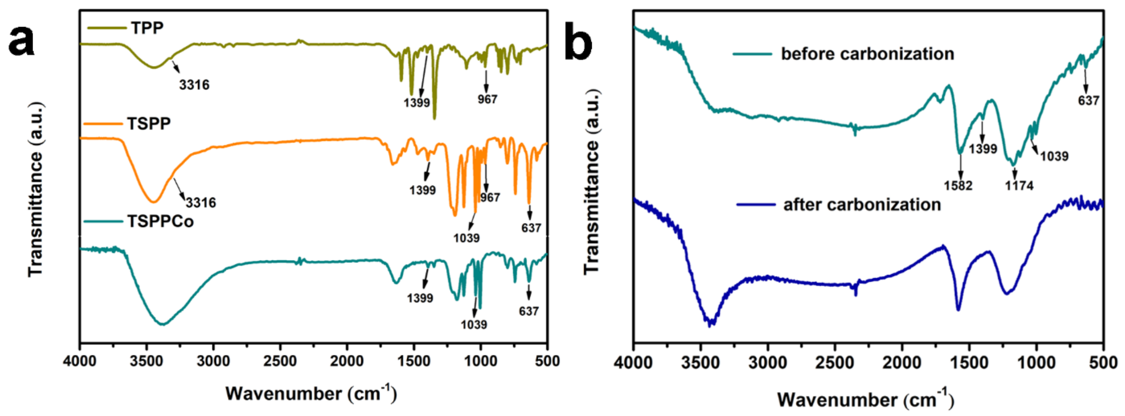

Fig. S4 a FTIR spectra of TPP, TSPP and TSPPCo. **b** FTIR spectra of Co_9S_8/NSG-700 before and after carbonization

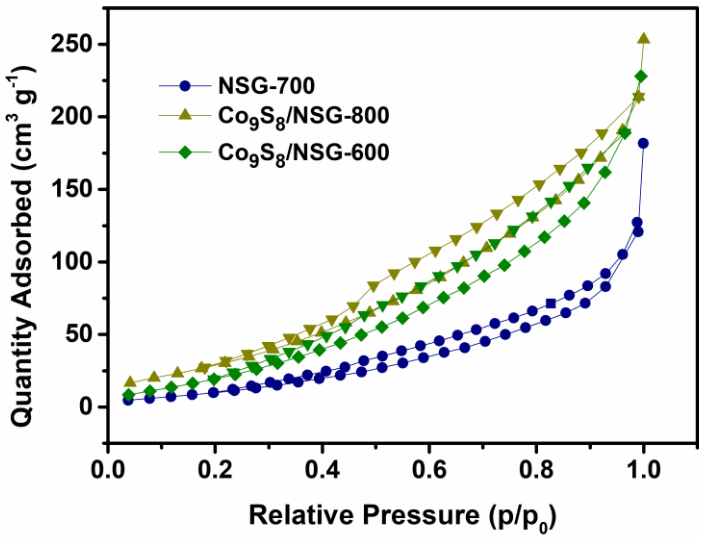

Fig. S5 Nitrogen adsorption and desorption isotherms of NSG-700, Co_9S_8/NSG-600, and Co_9S_8/NSG-800

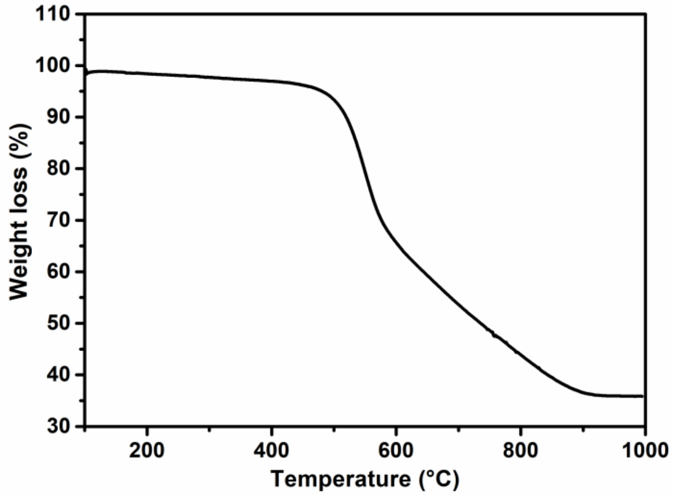

Fig. S6 TG curve of Co_9S_8/NSG-700

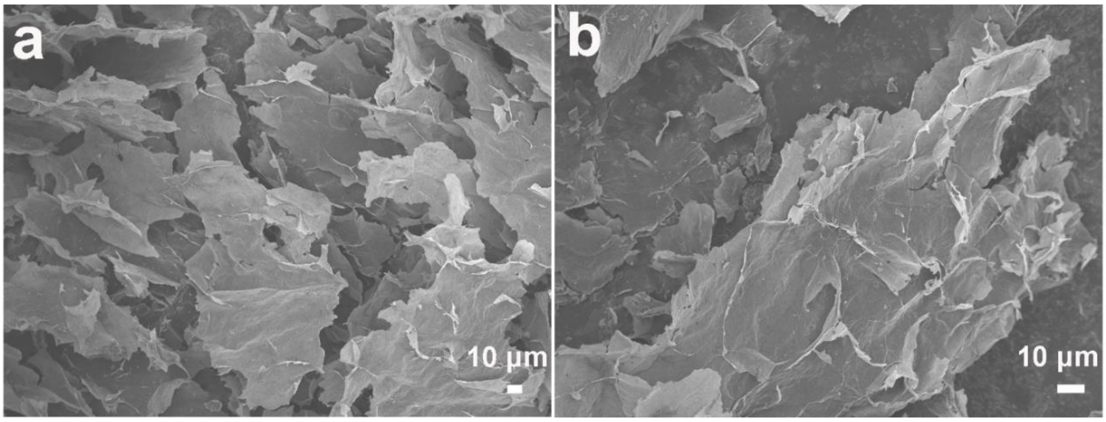

Fig. S7 SEM images of **a** Co_9S_8/NSG-600 and **b** Co_9S_8/NSG-800

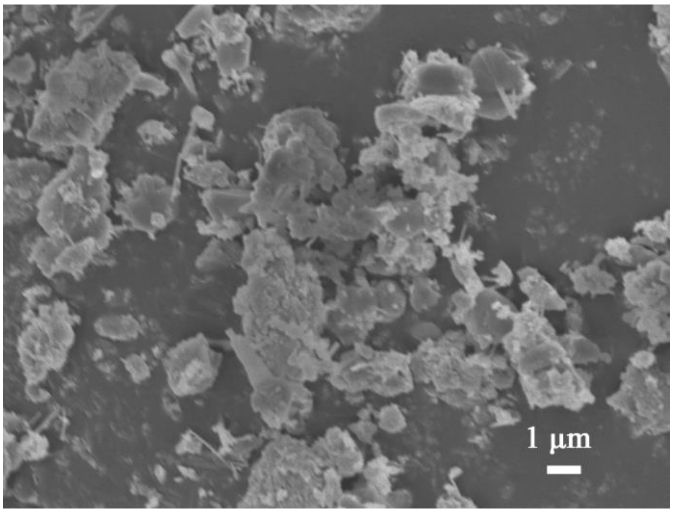

Fig. S8 SEM image of Co_9S_8/C-700

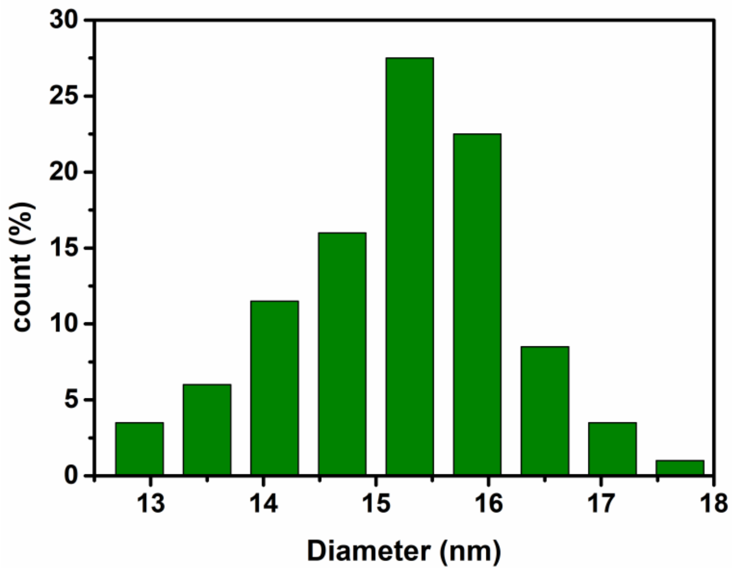

Fig. S9 Particle size distribution of Co_9S_8/NSG-700

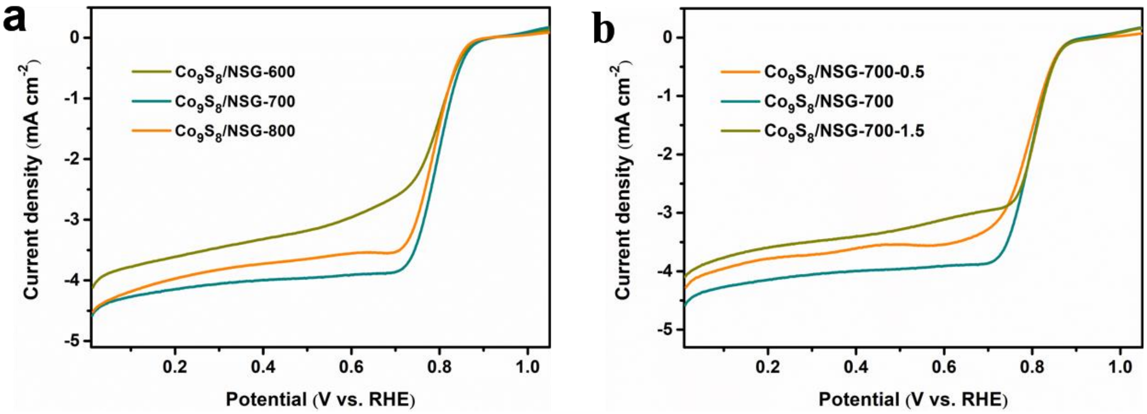

Fig. S10 LSV curves of **a** Co_9S_8/NSG-600, Co_9S_8/NSG-700, Co_9S_8/NSG-800 and **b** Co_9S_8/NSG-700-0.5, Co_9S_8/NSG-700, Co_9S_8/NSG-700-1.5

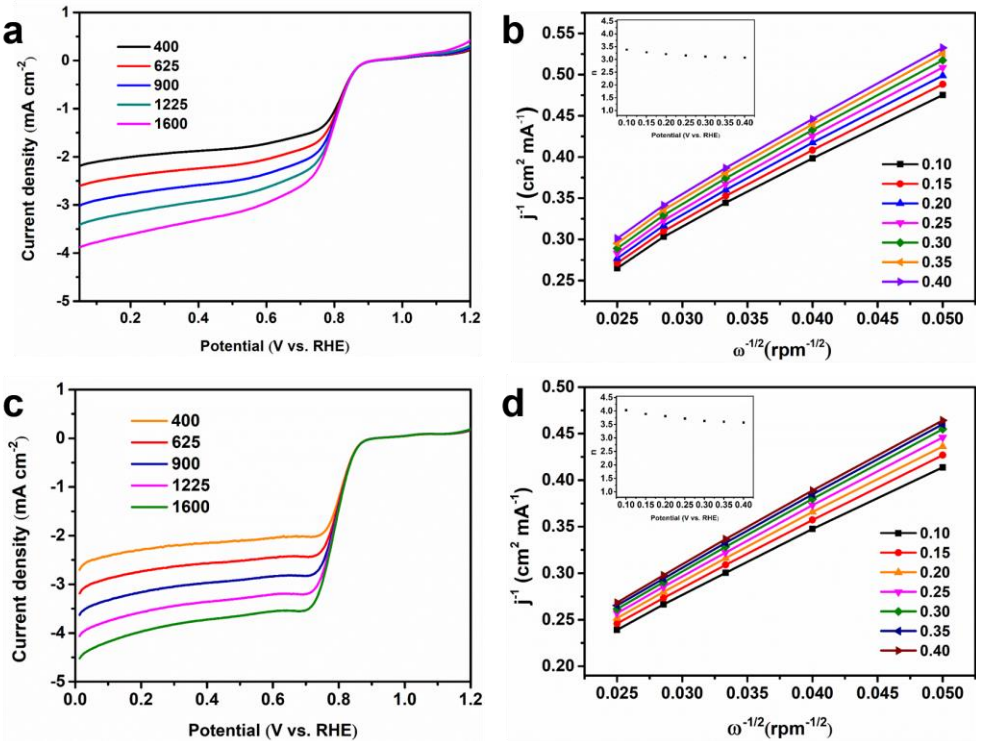

Fig. S11 a LSV curves of Co_9S_8/NSG-600 at different rotating rates. **b** K-L plots and the electron transfer number (insert) obtained from RDE results of Co_9S_8/NSG-600. **c** LSV curves of Co_9S_8/NSG-800 at different rotating rates. **d** K-L plots and the electron transfer number (insert) obtained from RDE results of Co_9S_8/NSG-800

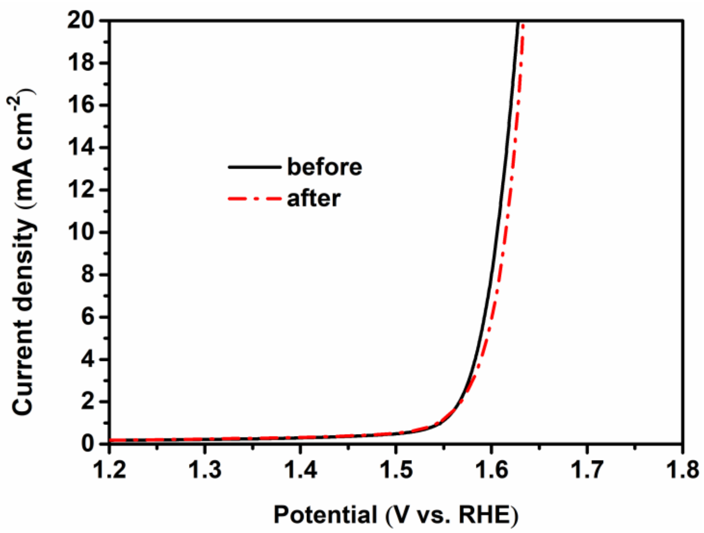

Fig. S12 OER polarization curves of Co_9S_8/NSG-700 before and after a continuous 2000-cycle CV scan

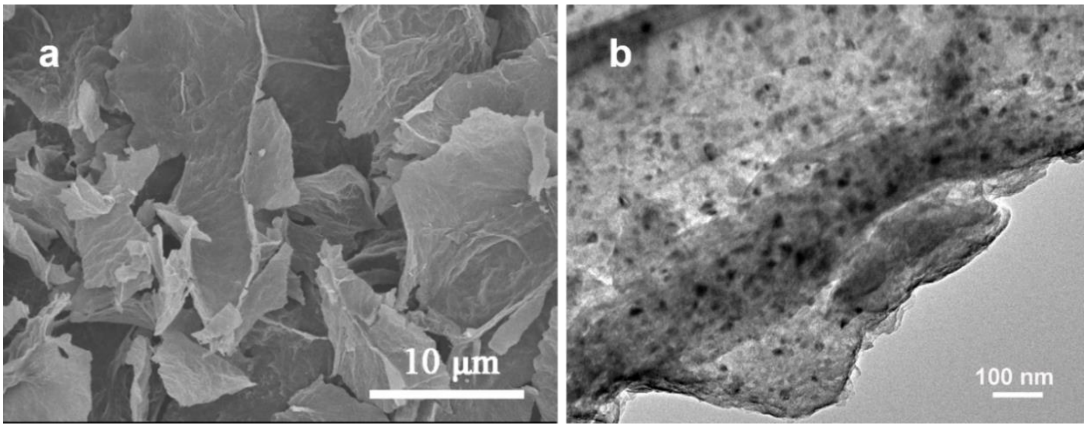

Fig. S13 a SEM image and **b** TEM image of Co_9S_8/NSG-700 after OER test

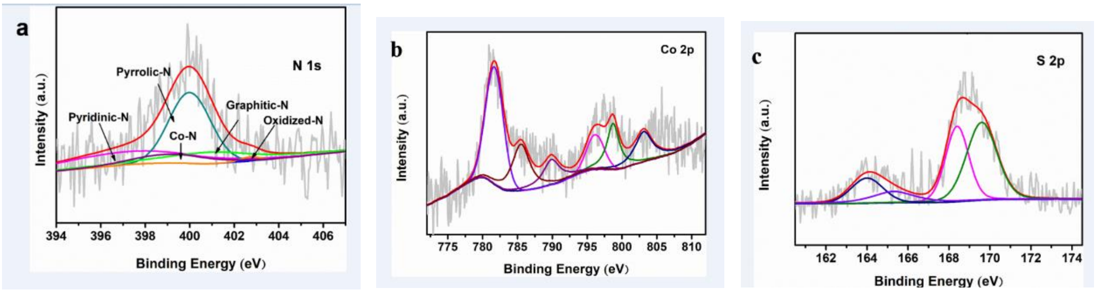

Fig. S14 High resolution spectra: **a** N 1s, **b** Co 2p and **c** S 2p of Co_9S_8/NSG-700 after the OER catalytic process

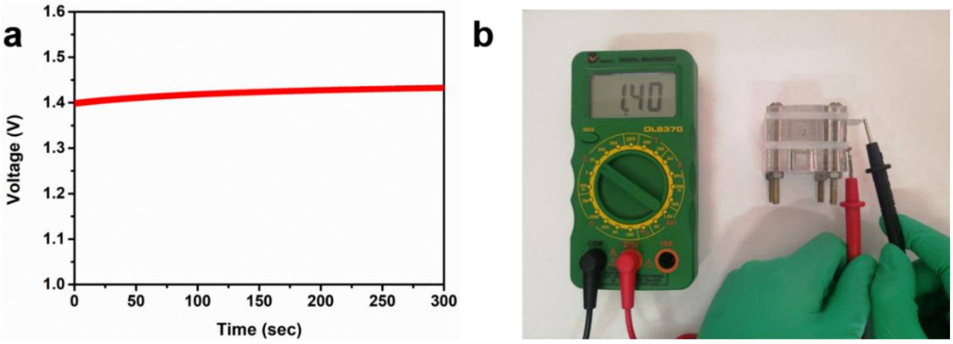

Fig. S15 a Open-circuit plots of assembled rechargeable Zn-air battery of Pt/C-RuO_2 catalysts. **b** Photograph of open-circuit potential

Table S1 Elemental contents of C, O, N, S and Co in the Co_9S_8/NSG-700 before and after OER test determined by XPS analysis

Catalyst	C (at%)	S (at%)	N (at%)	O (at%)	Co (at%)
Co_9S_8/NSG-700 before OER	89.38	2.16	2.89	5.12	0.45
Co_9S_8/NSG-700 after OER	72.2	2.07	3.57	21.65	0.51

Table S2 A survey of the catalytic performance of various bifuctional electrocatalysts

Catalysts	Loading (mg cm^{-2})	ORR		OER	ΔE	References
		E_{onset} (V)	$E_{1/2}$ (V)	$E_{j=10}$ (V)	($E_{j=10}$-$E_{1/2}$) (V)	
Co_9S_8/NSG	0.25	0.92	0.79	1.61	0.82	**This work**
Co_9S_8/NSPG	0.283	--	0.8	1.51	0.82	**ACS Sustainable Chem. Eng.** 2017, 5, 9848-9857
Co_9S_8@NSCM	0.15	0.97	0.81	1.60	0.79	**Nanoscale** 2018, 10, 2649-2657
Co-N-pCNs	0.25	0.96	0.80	1.63	0.83	**ChemCatChem.** 2017, 9, 1601-1609
N-GCNT/FeCo	0.2	1.03	0.92	1.73	0.81	**Adv. Energy Mater.** 2017, 7, 1602420
Co_3O_4/NPGC	0.2	0.97	0.84	1.68	0.84	**Angew. Chem. Int. Ed.** 2016, 55, 4977-4982
NiCo/PFC	0.13	0.92	0.79	1.63	0.84	**Nano Lett.** 2016, 16, 6516-6522
CoS_x@PCN/rGO	0.408	--	0.78	1.57	0.79	**Adv. Energy Mater.** 2018, 8, 1701642
$CuCo_2S_4$ NSs	0.2	0.90	0.70	1.52	0.82	**Nanoscale** 2018, 10, 6581-6588

Notes: ORR and OER Data reported in these works are normalized into reversible hydrogen potential (RHE).

Table S3 A survey of the performance of Zn-air batteries with various electrocatalysts

Catalysts	Loading (mg cm^{-2})	Peak Power (mW cm^{-2})	Open circuit potential (V)	References
Co_9S_8/NSG	1.0	72.4	1.42	**This work**
N-GRW	0.5	65	1.46	**Sci. Adv.** 2016, 2, e1501122
N-CN9	1.0	41	1.13	**Electrochim. Acta.** 2017, 247, 1044-1051
NPMC	0.5	55	1.48	**Nat. Nanotech.** 2015, 10, 444-452
S, N-Fe/N/C-CNT	1.25	102.7	1.35	**Angew. Chem. Int. Ed.** 2017, 56, 610-614
N8-VA-CNTs/GF	1.3	67	1.45	**J. Mater. Chem. A** 2017, 5, 2488-2495
c-$CoMn_2$/C	2.0	79	--	**Nat. Commun.** 2015, 6, 7345
NiFeO@MnO_x	0.25	81	1.32	**ACS Appl. Mater. Interfaces** 2017, 9, 8121-8133
Fe@C-NG/NCNT	1.0	101.3	1.37	**J. Mater. Chem. A** 2018, 6, 516-526

S3 References

[S1] W.S. Hummers, R.E. Offeman. Preparation of graphitic oxide. J. Am. Chem. Soc. **80**(6), 1339 (1958). https://doi.org/10.1021/ja01539a017

[S2] A.D. Adler, F.R. Longo, J.D. Finarelli, J. Goldmacher, J. Assour, L. Korsakoff. A simplified synthesis for meso-tetraphenylporphine. J. Org. Chem. **32**(2), 476 (1967). https://doi.org/10.1021/jo01288a053
